# Management of Pediatric Febrile Seizures

**DOI:** 10.3390/ijerph15102232

**Published:** 2018-10-12

**Authors:** Daniela Laino, Elisabetta Mencaroni, Susanna Esposito

**Affiliations:** Pediatric Clinic, Department of Surgical and Biomedical Sciences, Università degli Studi di Perugia, Piazza L. Severi 1, 06132 Perugia, Italy; daniela.laino@hotmail.it (D.L.); elisabetta.mencaroni@ospedale.perugia.it (E.M.)

**Keywords:** convulsion, epilepsy, febrile seizure, fever, pediatric neurology

## Abstract

Febrile seizures (FS), events associated with a fever in the absence of an intracranial infection, hypoglycaemia, or an acute electrolyte imbalance, occur in children between six months and six years of age. FS are the most common type of convulsions in children. FS can be extremely frightening for parents, even if they are generally harmless for children, making it important to address parental anxiety in the most sensitive manner. The aim of this review was to focus on the management of FS in the pediatric age. An analysis of the literature showed that most children with FS have an excellent prognosis, and few develop long-term health problems. The diagnosis of FS is clinical, and it is important to exclude intracranial infections, in particular after a complex FS. Management consists of symptom control and treating the cause of the fever. Parents and caregivers are often distressed and frightened after a FS occurs and need to be appropriately informed and guided on the management of their child’s fever by healthcare professionals. Due to the inappropriate use of diagnostic tests and treatments, it is extremely important to improve the knowledge of pediatricians and neurologists on FS management and to standardize the diagnostic and therapeutic work-up.

## 1. Introduction

Febrile seizures (FS) are seizures or convulsions that occur in children between six months and six years of and are triggered by fever [1]. FS are the most common type of convulsions in children. Their prevalence is approximately 3%–4% in white children, 6%–9% in Japanese children, and 5%–10% in Indian children [2]. FS can be extremely frightening for parents, even if they are generally harmless for children, making it important to address parental anxiety in the most sensitive manner [3].

The exact causes of FS are still unknown, although some studies indicate a possible association with environmental and genetic factors [4]. Fever is a normal response to infection, and the release of high levels of cytokines during a fever may alter normal brain activity, triggering seizures [5]. As demonstrated by earlier studies, the risk factors for FS are male gender, a family history of FS, an elevated peak body temperature, certain underlying causes of the fever, prenatal and natal complications, low serum calcium, sodium or blood sugar, microcytic hypochromic anemia, and iron and zinc deficiencies [6,7]. Indar Kumar et al. hypothesized that optimizing the reducible risk factors of FS could decrease the incidence [6]. Other studies showed that FS are associated with a polygenetic inheritance, even if an autosomal dominant inheritance pattern of a defined “febrile seizure susceptibility trait” has been identified in some families. Finally, mutations in the gene that encodes for sodium channels and the γ-aminobutyric acid A receptor may play a role in the development of FS [8]. The most frequent infections associated with FS in children are chickenpox, influenza, middle ear infections, upper and lower airway infections (such as tonsillitis, pneumonia, bronchitis and sinusitis), tooth infections, and gastroenteritis (especially those caused by rotavirus) [5,9]. Guedj et al. estimated that the risk of bacterial meningitis in children aged 6–11 months with a first simple FS was extremely low and encouraged the development of new guidelines to limit routine lumbar punctures in these children [10,11]. Prior work also reported that a cerebral abscess may initially present with FS and, even if rare, should therefore be considered in the differential diagnosis [12].

Considering the frequent inappropriate prescription of diagnostic examinations and the abuse of drugs in children with FS, the aim of this review is to focus on the management of FS in the pediatric age. PubMed was used to search for all the studies published over the last 20 years using the key words: “febrile seizures” and “children” or “infant” or “paediatric” or “pediatric”. More than 3000 articles were found, but only those published in English or providing evidence-based data were included in the evaluation.

## 2. Epidemiology and Clinical Features

FS have a prevalence of 2%–5% in children in Western Europe and the United States, and the peak age of onset is 18 months [9,13]. Children aged 12–30 months represent 50% of all children with FS, while the proportion of children who experience a first episode of FS after four years of age is low (6%–15%) [9,13]. Children of all ethnic groups may present with FS, but there is a higher prevalence in some ethnic groups, in particular Guamanians (14%), Japanese (6%–9%), and Indians (5%–10%) [13,14].

FS usually occur when the child’s temperature is more than 38 °C, although children may develop seizures at any point during a febrile disease and may only develop a fever after their seizure. Typical signs and symptoms of FS include a loss of consciousness, difficulty breathing, pallor or turning blue, foaming at the mouth, eyes rolling to the back of the head, a fixed gaze, generalized or focal twitching, and jerking of the arms and legs. After a seizure, children may be irritable, confused or drowsy but will completely recover after approximately 30 min [14,15,16,17]. There are two main types of FS: simple FS, which make up 70% of all FS and generally have no long-term neuro-developmental consequences, and complex FS. The characteristics of simple and complex FS are described in Table 1. Febrile Status Epilepticus (SE) is defined as an FS that lasts more than 20 min and generally requires the administration of anticonvulsants to interrupt it.

Moreover, in 1997 genetic epilepsy with febrile seizures plus (GEFS+) was described [18]. GEFS+ is a familial epilepsy syndrome in which affected individuals within a family typically have a variety of epilepsy phenotypes, varying from simple febrile seizures and febrile seizures plus with a good outcome to severe epileptic encephalopathies. GEFS+ is a rare condition and its prevalence is unknown. GEFS+ is inherited in an autosomal dominant pattern [19]. Mutations in several genes can cause GEFS+. The most commonly associated gene is *SCN1A* [20]. Because the clinical manifestations are so varied, even among family members, other genes and environmental factors help determine the severity of the condition.

## 3. Diagnosis

When a child with FS presents to the Emergency Department (ED), it is important, above all, to collect a detailed and accurate history and to perform a complete clinical evaluation, including a neurologic examination, to rule out secondary causes of convulsions [13,14,17,21]. The differential diagnosis of FS is described in Table 2 and includes rigors, febrile delirium, febrile syncope, breath holding attacks, reflex anoxic seizures, evolving epilepsy syndromes, and central nervous system infections. Generally, a history is collected from the parents or caregivers and should include the nature and duration of the convulsions, the presence and duration of the post-ictal phase, recent infectious diseases or fevers, recent use of antibiotic therapy, other associated symptoms, immunization and vaccination history, a history of previous episodes of FS or a diagnosis of epilepsy, other neurologic conditions and diseases, a family history of FS, epilepsy, or neurologic diseases, the use of antipyretics, and the need for rescue anticonvulsants to interrupt seizures, such as diazepam or midazolam [4]. The clinical evaluation should focus on identifying the infection causing the fever.

If still convulsing, the child needs emergency stabilization using the ABCDE approach (airway, breathing, circulation, disability, and exposure/examination, plus blood glucose check) [3,22] and the seizure should be stopped with antiepileptic drugs as soon as possible (see management). After stabilization, vital signs should be recorded: temperature, heart and respiratory rate, capillary refill time, and blood glucose [16,17]. In young children, the signs and symptoms of intracranial infections, such as meningitis or encephalitis, may be very subtle, and these infections must be ruled out as soon as possible [14,22,23,24]. Brain abscesses are a rare entity in pediatric patients and occur in children younger than 15 years of age in 25% of cases, with a peak incidence at 4–7 years of age. Seizures, focal neurologic deficits and an altered mental status are present in 25%–50% of patients, but symptoms may also not be evident. Brain magnetic resonance imaging (MRI) is the first diagnostic modality of choice, and a lumbar puncture is not recommended if the MRI visualizes the abscess. Treatment consists of antibiotic therapy and surgical drainage of the abscess. The most frequent organisms that cause brain abscesses are streptococci, staphylococci, and enteric bacteria, but of greater concern is the role of community-acquired methicillin-resistant *Staphylococcus aureus* (MRSA) over the last decade [11].

Furthermore, it is necessary to differentiate between a first FS and the first episode of afebrile or epileptic convulsions, and a clear history of fever, either before or soon after FS, should be identified [22]. Testing should be performed in children who present with the signs and symptoms of a serious illness or an intracranial infection (pneumonia or meningitis/encephalitis), but is not necessary in children aged over one year who have a clear focus of infection, are fully immunized, and present with a simple FS [24]. In children aged less than one year who present with a first episode of complex FS or have symptoms suggestive of an intracranial infection, further investigations should be considered [9], including labs such as a full blood count, C-reactive protein, urea, calcium, magnesium, glucose, and electrolyte levels, and blood cultures if bacterial sepsis is suspected; urine dipstick and culture tests; chest X-rays; stool culture tests; and a lumbar puncture (this test should not be performed soon after a FS because in the post-ictal phase, it is difficult to identify a raised intracranial pressure). Naric et al. have demonstrated the role of multiplex PCR analysis in the identification of several causative viruses in children with febrile seizures and its potential for facilitating the risk stratification of these patients to reduce the unnecessary use of antibiotics in the future [25]. Furthermore, Remick et al. showed that hypoglycaemia was a rare finding in the prehospital or ED setting in children presenting with FS, and testing the blood glucose levels in this population had a low benefit and minimal clinical significance. They suggested that such tests should instead be performed on an actively seizing patient after the first dose of benzodiazepines or in a child with a persistently altered mental status [26]. Computed tomography (CT), MRI, electroencephalography (EEG), or a combination of these may be considered in children with a history of complex or recurrent FS or who present with neurological abnormalities to rule out the presence of neurologic conditions. After a FS in a healthy child with a clear source of infection, an EEG is not recommended.

In cases in which an EEG is performed, the EEG should be done at least 48 h after the FS to avoid confusing post-ictal electrical activities with abnormal electrical activities [14,27,28]. In addition, Harini et al. found that EEG slowing was not a significant predictor of epilepsy. Thus, early EEG abnormalities after a first complex FS are unlikely to identify patients at risk of epilepsy. There are likely to be multiple factors that influence the occurrence of EEG abnormalities, such as the age of the patient, the timing of the EEG, and genetic syndromes [29]. Repetition of this test is not necessary in children who present with recurrent episodes of simple FS and have a clear source of infection, but it is important to accurately determine the cause of the infection and to manage it in the most appropriate way [19].

## 4. Management

A child with a simple FS should not be hospitalized if he or she is in a good clinical condition and if the source of the infection is clear. The child can be discharged after a period of observation in the ED, preferably six hours after the episode. Most FS episodes are short-lived and self-terminating and do not require long-term treatment with antiepileptic drugs [27]. In a child who is still convulsing at presentation to the ED, indications for giving antiepileptic drugs are seizures lasting more than 5 min, febrile SE, and recurrent seizures. In the evaluation of a child with FS, it is important to recognize red flags, which are useful in deciding if further management is required (see Table 3). The National Institute for Health and Care Excellence (NICE) traffic-light system is helpful in identifying the signs and symptoms that predict the risk of a serious illness [17]. Hospitalization for observation is necessary when a child presents with red flag signs and symptoms, the seizure is prolonged, a complex FS occurs, residual neurological findings (i.e., Todd’s paresis) are present, a serious infection is suspected, the source of infection is not clearly determined, the child’s age is less than 18 months, there is a risk of seizure recurrence, and the parents or caregivers are not able to provide regular monitoring soon after the FS [9,17,22].

In the acute phase, treatment is directed at identifying the underlying cause of the fever and at its symptomatic management. It is important to ensure adequate hydration by encouraging the child to drink, and paracetamol or ibuprofen can be administered to relieve discomfort caused by the infection [14,17]. NICE does not recommend using paracetamol and ibuprofen together because the clinical benefit of using them together is small, it increases the risk of drug administration errors and overdoses, and it sends an incorrect message to parents [17,30,31]. Several trials have demonstrated that antipyretic drugs do not reduce the risk of FS recurrence, and therefore, attempting to reduce the child’s temperature is not recommended [13,14,16,17,20]. Parents and caregivers should be made aware that the rationale for the administration of antipyretic drugs is to relieve the discomfort caused by the infection, not to reduce the risk of FS [9,16]. Table 4 shows a list of drugs commonly used in the Emergency Room for children presenting with FS. In the case of bacterial, febrile infections, such as tonsillitis, otitis media, or pneumonia, antibiotics should be administered.

Long-term antiepileptic drugs are not generally prescribed as prophylaxis for FS, as it has been demonstrated that they do not reduce the risk of developing epilepsy, and their potential side effects outweigh their potential benefits [19,24,32,33,34].

On some occasions, benzodiazepines, such as rectal diazepam or buccal midazolam, can be prescribed for use at home as a rescue therapy to stop seizures [4]. Benzodiazepines can be used in children who present with frequent FS in a short period or for FS that last more than 15 min, if antiepileptic drugs have previously been required to stop the seizures [18,19,33]. A recent study by Offringa et al. reviewed the effects of antiepileptics, antipyretics, and zinc in children with FS. They concluded that neither continuous nor intermittent treatment with zinc, antiepileptics, or antipyretics is recommended for children with FS. Considering that FS can be frightening to witness, parents and families should be supported with adequate contact details for medical services and information on FS recurrence, first aid management and, most importantly, the benign nature of the phenomenon [35].

Several studies have recently addressed the management of FS in the Emergency room. They all concluded that there is a need for a standardized diagnostic work-up to improve the cost/benefit ratio of FS management [36,37,38].

## 5. Prognosis

Clinicians and parents/caregivers are often concerned about the recurrence of FS, particularly about the risk of the onset of epilepsy. Simple FS may slightly increase the risk of developing epilepsy, but have no adverse effects on behavior, scholastic performance, or neurocognition [4]. The risk of developing epilepsy is increased further in children with a history of complex FS [4]. One third of children who present with one FS will present with a second episode during a future febrile disease. Risk factors for the recurrence of FS are a positive family history of FS, a first FS before 18 months of age, the occurrence of a first FS less than one hour after the start of a fever, and FS at a body temperature of less than 38 °C [2,4,13,21]. Febrile seizures will recur in 4% of children with no risk factors but in 75% of the children with previously described risk factors [13,21]. It is important to know the risk factors for FS recurrence to counsel the child’s parents or caregivers and to administer rescue antiepileptics to children with a strong risk of recurrence.

## 6. Conclusions

FS are the most frequent type of seizures in pediatric patients. Most children have an excellent prognosis, and few develop long-term health problems. The diagnosis of FS is clinical, and it is important to exclude intracranial infections, in particular after a complex FS. Management consists of symptom control and treating the cause of the fever. Parents and caregivers are often distressed and frightened after a FS occurs and need to be appropriately informed on the usually favorable prognosis as well as guided by healthcare professionals on the management of their child’s fever and acute phase of FS. In order to avoid the abuse of diagnostic tests and treatments, pediatricians and neurologists should be appropriately informed on FS management.

## Figures and Tables

**Table 1:** Clinical characteristics of simple and complex febrile seizures (FS).

Simple	Complex
Generalized tonic-clonic seizures without focal features Seizures last less than ten minutes Seizures spontaneously resolve There is no recurrence within 24 h	There are focal features in which, for example, only one side of the body is involved Seizures last for more than ten minutes Two or more seizures occur within 24 h Full recovery is not observed after one hour There are post-ictal neurologic consequences There is a short period of paralysis, defined as Todd’s paralysis, after the seizure Febrile SE develops Anticonvulsant drugs may be required to interrupt the seizure

**Table 2:** Differential diagnosis of febrile seizures (FS).

Rigors: shaking without a loss of consciousness
Febrile delirium: acute and transient confusion associated with a high fever
Febrile syncope
Breath holding attacks: children voluntarily hold their breath and may transiently lose consciousness
Reflex anoxic seizures: children suddenly become limp because of painful events or shock
Evolving epilepsy syndrome: fever triggers seizure episodes
Central nervous system infections: meningitis, encephalitis, and brain abscesses

**Table 3:** Red flag signs and symptoms in a child presenting with febrile seizures (FS).

The child presents with complex FS
Meningeal signs are observed: a positive Kernig’s sign and/or a positive Brudzinski sign and/or neck stiffness
Altered level of consciousness for more than one hour after interruption of the FS
Evolving non-blanching rashes in an unwell child
Bulging anterior fontanelle
Tachycardia out of proportion with body temperature, or tachycardia that persists even after the normalization of body temperature
Signs of moderate to severe respiratory distress, such as tachypnea, grunting, low oxygen saturation (<92% on air), and chest wall recessions

**Table 4:** Drugs commonly used for children with febrile seizures (FS) who present to the Emergency Room.

Name	Dosage	Administration Route	Frequency	Maximum Dosage	When Used
Paracetamol	15 mg/kg	Oral, rectal or intravenous (IV) during resuscitation	Every four to six hours	Five within 24 h	For pyrexia in children with FS
Ibuprofen	5–10 mg/kg	Oral	Every six to eight hours	Four within 24 h	For pyrexia in children with FS unless they are dehydrated
Diazepam	0.25 mg/kg 0.5 mg/kg	IV or intraosseous Rectal	A second dose may be given ten minutes after the first	Only two doses of benzodiazepines are to be used, regardless of the agent selected and if they are administered alone or in combination	For an actively convulsing child whose seizures have lasted more than five minutes
Lorazepam	0.1 mg/kg	IV	A second dose may be given ten minutes after the first	Only two doses are to be used	For an actively convulsing child whose seizures have lasted more than five minutes
Midazolam	0.15–0.2 mg/kg	IV	A second dose may be given 10 min after the first	Only two doses are to be used	For an actively convulsing child whose seizures have lasted more than five minutes
0.9% sodium chloride solution	20 mL/kg	IV	During resuscitation	More than two doses are rarely required	In children with shock, for example, in febrile illness due to gastroenteritis
